# Time is Blood: The Impact of Diagnostic Delays on Acquired Hemophilia A

**DOI:** 10.7759/cureus.22048

**Published:** 2022-02-09

**Authors:** Michael Fragner, Bailey Imbo, Jared Hobson, Jonathan C Roberts, Anita Rajasekhar, Michael D Tarantino, Jason Morell, Amar H Kelkar

**Affiliations:** 1 Physical Medicine and Rehabilitation, Saint George’s University School of Medicine, True Blue, GRD; 2 Orthopedic Surgery, Saint George’s University School of Medicine, True Blue, GRD; 3 Radiation Oncology, Saint George’s University School of Medicine, True Blue, GRD; 4 Hematology, Bleeding & Clotting Disorders Institute, Peoria, USA; 5 Division of Hematology and Oncology, University of Florida College of Medicine, Gainesville, USA; 6 Department of Pharmacy, Baptist Health South Florida, Miami, USA; 7 Division of Stem Cell Transplantation and Cellular Therapies, Dana-Farber Cancer Institute/Brigham and Women's Cancer Center/Harvard Medical School, Boston, USA

**Keywords:** bleeding, diagnostic delays, aha, acquired hemophilia a, factor viii inhibitor

## Abstract

Background and objective

Acquired hemophilia A (AHA) is an uncommon autoimmune bleeding disorder caused by the formation of neutralizing antibodies against endogenous factor VIII (FVIII). Delays between the onset of symptoms and the correct diagnosis of the condition lead to poor outcomes and a higher mortality rate. In this study, we aimed to analyze the impact of delays in diagnosis on AHA patients.

Methods

We conducted a retrospective study at a single hospital system between March 1, 2010, and January 17, 2017, which included six patients meeting the criteria for AHA diagnosis.

Results

Initial analysis revealed a median age of 79.5 years and a median time to diagnosis from the onset of bleeding of 14 days. Among the six patients, three had cancer (bladder, renal, and prostate) and three had unknown etiologies. One of the patients died prior to the initiation of a bypassing agent. The remaining five patients received recombinant FVIIa (NovoSeven®, Novo Nordisk, Bagsværd, Denmark), and two of those five required a second-line bypassing agent, recombinant porcine sequence FVIII (Obizur®, Takeda Pharmaceutical, Tokyo, Japan) for refractory bleeding. All five patients achieved hemostasis; however, three died within a year, and none of the patients survived for five years. Four of these five patients died directly from bleeding complications.

Conclusions

Based on our study findings and review of the literature, we propose an algorithm to potentially aid in the early diagnosis and treatment of AHA in emergency and non-specialized settings.

## Introduction

Acquired hemophilia A (AHA) is an autoimmune disorder characterized by the formation of neutralizing autoantibodies against endogenous factor VIII (FVIII) [1]. The formation of these autoantibodies can be triggered by underlying risk factors such as malignancy, infection, concurrent autoimmune disease, lymphoproliferative disease, advanced age, pregnancy, as well as iatrogenic causes such as surgery, but is also reported to be idiopathic in 51.9% of cases [1]. Several registry studies and clinical trials have prospectively followed patients with AHA in the last 20 years, with the largest being the European Acquired Haemophilia (EACH2) registry [1-6]. In the EACH2 cohort, AHA was typically found in older patients, with a reported median age of 73.9 years, a male predominance in approximately 57% of non-pregnancy-related cases, and a lack of personal or family history of bleeding [1]. Cases of AHA are typically identified due to unexplained, often spontaneous bleeding diathesis, hematoma formation, or an isolated prolongation of activated partial thromboplastin time (aPTT) [1]. Bleeding symptoms in AHA tend to manifest as ecchymosis, hematomas, hematuria, and mucosal bleeding, with hemarthrosis and intracranial bleeding being rare events [7].

Evaluation for suspected AHA should include aPTT testing, followed by simultaneous testing with an aPTT mixing study, FVIII activity, and confirmatory tests (Bethesda assay or ELISA) [8]. Modern treatment regimens for AHA include bypassing agents to circumvent neutralized FVIII in the coagulation cascade, and immune suppression therapy (IST) [8]. Bypassing agents include recombinant activated factor VII [5], activated prothrombin complex concentrate [8,9], and recombinant porcine sequence FVIII [6]. The goal of IST is to eradicate the autoantibody. Typical agents include corticosteroids, cyclophosphamide, and rituximab [8]. The use of IST has resulted in remission rates of 60-90% in various studies [3]. However, mortality rates in patients with AHA have been reported to be as high as 28-42% due to direct complications from bleeding, as well as adverse reactions including infection from immune suppression [3].

We present a retrospective study of patients with AHA conducted at a large healthcare system. We analyze the patient demographics, treatment decisions, patient outcomes, and the cause of diagnosis delays and their impact on patients.

## Materials and methods

Patient data

This was a single-center, retrospective study of patients meeting the diagnostic criteria for AHA within the OSF Healthcare System in Illinois, between March 1, 2010, and January 17, 2017. The data from the Healthcare Analytics department at OSF Healthcare was utilized to identify patients based on laboratory tests.

Patients were initially identified based on prior testing for a factor VIII inhibitor (“Factor 8 Assay” and “F8 Inhibitor”) within the study period. Patients were included based on either a positive FVIII mixing study with Bethesda assay or a factor VIII inhibitor ELISA. Patients were excluded if they were neonates (aged zero to four weeks), prisoners, or anyone with congenital factor VIII deficiency. The study design was presented to the University of Illinois College of Medicine at Peoria Internal Review Board and granted exempt status; 1,931 patients were initially identified, of which six patients met the aforementioned criteria for the diagnosis of AHA.

Statistical analyses

This was an observational study with the primary aim of describing the presenting characteristics, bleeding and survival outcomes, and delays in the diagnosis of patients with AHA. Time to diagnosis was calculated in days, as the time from the first presentation to a medical facility until the first positive screening or confirmatory testing. The secondary aim was a description of the treatment decisions for patients with AHA in the era of multiple bypassing agents. Descriptive statistics were calculated using Microsoft Excel (Microsoft Corporation, Redmond, WA).

## Results

Demographics

The study population of six patients diagnosed with AHA had a median age of 79.5 years (range: 61-90 years). Five of the six patients were males. Four of the patients were White and the remaining two patients were Black. All six patients had an extensive medical history including hypertension, malignancy, and autoimmune disease. The etiology of AHA was suspected to be an underlying malignancy in four of the six patients and autoimmune disease in one patient. The remaining one case was suspected to be idiopathic. The demographic and medical characteristics of the six patients are presented in Table 1.

**Table 1:** Demographics, presenting complaints, and etiology of patients with AHA AHA: acquired hemophilia A; MRSA: methicillin-resistant *Staphylococcus aureus*; GI: gastrointestinal; GIST: gastrointestinal stromal tumor

#	Age, years	Race	Sex	Comorbidities	Bleeding symptoms	Suspected etiology
1	82	White	Male	Hypertension, coronary artery disease, chronic systolic heart failure, atrial fibrillation, chronic obstructive pulmonary disease, osteoarthritis, benign prostatic hyperplasia, hyperlipidemia, former smoker (37.5 pack-years), bladder carcinoma	Gross hematuria	Bladder cancer
2	64	White	Male	Hypertension, diabetes mellitus type II, hyperlipidemia, chronic obstructive pulmonary disease, former smoker (50 pack-years)	Left-arm bruising, left-elbow hemarthrosis	Unknown
3	61	Black	Male	Hypertension, end-stage renal disease, paroxysmal atrial fibrillation, stroke, chronic diastolic heart failure, seizure disorder, splenectomy, current smoker (35 pack-years), renal mass	Bruising, post-procedural bleeding (following hemodialysis catheter placement)	Suspected renal cancer
4	77	White	Female	Hypertension, hypothyroidism, bullous pemphigoid, arthritis, former smoker (50 pack-years)	GI bleeding, hemorrhagic shock	Autoimmune disease
5	90	White	Male	Hypertension, hyperlipidemia, former smoker (30 pack-years)	Gross hematuria	Prostate cancer
6	83	Black	Male	Hypertension, diabetes mellitus type II, chronic kidney disease stage III, GIST tumor, acute deep vein thrombosis, former smoker (20 pack-years)	Upper GI bleeding, bleeding GIST tumor	GIST tumor

Presentation

All the patients presented with bleeding symptoms, and five of the six met the criteria for major bleeding per International Society on Thrombosis and Haemostasis (ISTH) criteria, as described in Table 1 and Table 2 [10]. Presenting complaints ranged from constitutional symptoms including fatigue to bleeding symptoms including bruising, hematuria, gastrointestinal (GI) bleeding, and post-procedural bleeding. Subsequent bleeding sites included the buttocks, upper and lower extremities joints and soft tissue, neck, and GI tract. Five of the six patients developed hematoma or hemarthrosis while under treatment. All but one patient required a blood transfusion, and two patients were put on massive transfusion protocols. The time to initial diagnosis could be determined in five of the six patients: median duration of 14 days (range 5-91 days), with diagnostic testing resulting after death in one patient. However, one patient with a delayed diagnosis time of 91 days was on anticoagulation (warfarin) for atrial fibrillation prior to the presentation, which acted as a confounder.

**Table 2:** Bleeding symptoms and outcomes in patients with AHA AHA: acquired hemophilia A; pRBC: packed red blood cells; MTP: massive transfusion protocol; N/A: not applicable; LLE: left lower extremity; RUE: right upper extremity

#	Major bleeding	Bleeding site	Time (days) from the onset of bleeding to diagnosis	Time (days) from the start of treatment to the resolution of bleeding
1	Yes (required 2U pRBC)	Right buttock, thigh, and leg	91	1
2	Yes (required 4U pRBC)	Left-elbow hemarthrosis	5	4
3	Yes (MTP)	Left-neck bleeding	10	Not reported
4	Yes (MTP)	GI bleeding	Death prior to results of confirmatory testing	N/A
5	No	LLE hematoma	14	20
6	Yes (required 5U pRBC)	Bleeding gastric mass, RUE hematoma	20	1

Laboratory testing

All six patients presented with a prolonged aPTT with a median value of 86.5 seconds (range: 75.9-139 seconds). The aPTT mixing study was performed in four patients with a median result of 51.5 seconds (range: 44-66.7 seconds). All six patients screened positive for FVIII inhibitors. Five of the six patients had a measured Bethesda assay titer, with a median value of 106.5 Bethesda units (range: 33-180 units), with one sample found insufficient to be measured. FVIII activity was measured in four patients with three patients showing results of less than 1% and one patient with 10%. Two patients (3 and 4) did not have their FVIII activity data collected. These laboratory values are summarized in Table 3.

**Table 3:** Laboratory testing results of patients with AHA ^1^Labs collected on admission to the hospital AHA: acquired hemophilia A; BU: Bethesda units; aPTT: activated partial thromboplastin time; Hb: hemoglobin; LA: lupus anticoagulant

#	FVIII inhibitor screen	Bethesda assay (BU)	FVIII level	Factor IX level	aPTT mixing study (seconds)	Hb (g/dL)^1^	aPTT (seconds)^1^	INR^1^	Fibrinogen (mg/dL)^1^	LA
1	Positive	106.5	<1%	Not collected	Not collected	8.3	82	1.2	Not collected	Not collected
2	Positive	Insufficient sample	10%	227%	66.7	6.6	83	1.1	640	Negative
3	Positive	70	Not collected	159%	45	5.4	97	1.2	Not collected	Negative
4	Positive	180	Not collected	Not collected	58	4.5	90	Not collected	Not collected	Negative
5	Positive	33	<1%	Not collected	Not collected	Not collected	75.9	Not collected	Not collected	Not collected
6	Positive	111	<1%	Not collected	44	6.4	139	1.1	482	Negative

Management

Five of the six patients received a bypassing agent, either as first-line or second-line therapy, and all five patients subsequently achieved hemostasis. Three patients required second-line therapy. Four patients received recombinant factor VIIa (NovoSeven®, Novo Nordisk, Bagsværd, Denmark) as first-line therapy and one patient received it as second-line therapy. One patient received desmopressin as first-line therapy. Two patients received porcine sequence recombinant FVIII (Obizur®, Takeda Pharmaceutical, Tokyo, Japan) as second-line therapy. Both of these patients were also included in a recent case series on porcine recombinant FVIII [11]. Prednisone was used in four cases, with two using a second immunosuppressant (cyclophosphamide or rituximab). One patient achieved hemostasis with a bypassing agent and was discharged without prednisone or IST. The FVIII inhibitor level was not tracked thereafter. None of the patients required a third-line treatment to achieve hemostasis. These management plans and bleeding outcomes are summarized in Tables 2, 4.

**Table 4:** Treatment plans for patients with AHA AHA: acquired hemophilia A; N/A: not applicable; IV: intravenous

#	First-line treatment	First-line dosage	Second-line treatment	Second-line dosage	Immune suppression therapy
1	Recombinant factor VIIa	2 doses (90 mcg/kg)	N/A	N/A	Rituximab (IV weekly), prednisone taper (starting 80 mg daily)
2	Recombinant factor VIIa	1 dose (90 mcg/kg) followed by 9 doses (48 mcg/kg every 6 hours)	Porcine recombinant factor VIII	1 dose (100 U/kg) followed by 3 doses (80 U/kg every 24 hours)	Prednisone, cyclophosphamide 50 mg daily, porcine recombinant factor VIII 80 U/kg daily
3	Desmopressin	Unknown	Recombinant factor VIIa	90 mcg/kg every 3 hours until bleeding stops	Prednisone 80 mg with taper
4	Died prior to the initiation of therapy	N/A	N/A	N/A	N/A
5	Recombinant factor VIIa, methylprednisolone	8 mg (every four hours for 4 days, followed by every 12 hours on discharge)	Porcine recombinant factor VIII	50 U/kg daily (every other day after 1 month)	Prednisone taper (starting at 60 mg daily)
6	Recombinant factor VIIa	2 mg daily	N/A	N/A	N/A

Remission and recurrence

Remission was achieved in four of the six patients, though inconsistencies in follow-up testing made it difficult to determine the time to eradication of the factor inhibitor and normalization of FVIII activity. All four patients achieving remission had a recurrence of low FVIII activity and three of those patients had a recurrence of bleeding. All four patients received subsequent bypassing agents including recombinant factor VIIa, porcine recombinant factor VIII, and activated prothrombin complex concentrate (FEIBA®, Takeda Pharmaceutical). The one patient receiving recombinant factor VIIa without bleeding received it as pre-procedural prophylaxis for a planned surgery. These findings are summarized in Table 5.

**Table 5:** Remission and recurrence management in patients with AHA AHA: acquired hemophilia A; N/A: not applicable

#	Time (days) to first remission (of factor inhibitor assay)	Time (days) to first remission (of FVIII activity)	Remission (eradication of inhibitor)	Procedural prophylaxis	Recurrence of low FVIII	Recurrence of bleeding	Management of recurrence
1	11	54	Yes	N/A	Yes	Yes	Recombinant factor VIIa (for short-term bleeding), porcine recombinant factor VIII (for prolonged bleeding)
2	Unknown	5	Yes	N/A	Yes	Yes	Recombinant factor VIIa, porcine recombinant factor VIII, activated prothrombin complex concentrate, aminocaproic acid
3	43	6	Yes	NovoSeven	Yes	No	NovoSeven (received as pre-procedural prophylaxis)
4	N/A	N/A	No	N/A	N/A	N/A	N/A
5	N/A	20	Yes	N/A	Yes	Yes	Recombinant factor VIIa (9 mg/dose)
6	N/A	N/A	No	N/A	N/A	N/A	N/A

Outcomes

All six patients were alive one week after the initial presentation. However, one patient developed hemorrhagic shock and died in the ICU without receiving hemostatic therapies or IST. Three of the six patients died within one year of the initial presentation. At the time of data collection in 2017, five of the six patients had died. Four of the five deceased patients had died from bleeding complications related to recurrence and the other patient had died from infection. These findings are summarized in Table 6.

**Table 6:** Outcomes in patients with AHA AHA: acquired hemophilia A; N/A: not applicable

#	48-hour survival	1-week survival	1-year survival	5-year survival	Status	Death related to bleeding
1	Yes	Yes	Yes	N/A	Alive	N/A
2	Yes	Yes	No	No	Deceased	Yes
3	Yes	Yes	Yes	No	Deceased	No
4	Yes	Yes	No	No	Deceased	Yes
5	Yes	Yes	Yes	No	Deceased	Yes
6	Yes	Yes	No	No	Deceased	Yes

## Discussion

Summary of findings

AHA is a rare blood disorder, which may lead to life-threatening bleeding if not diagnosed and treated in a timely manner [1]. Even though we had access to electronic patient data from one of the largest hospital systems in Illinois, we found only six confirmed cases of AHA in our nearly seven-year study period. Historically, the incidence of AHA has been reported at approximately 1.48 cases per million per year, and the paucity of cases identified within our study is consistent with its relative rarity [2]. Compared to the incidence of AHA in the general population, our study showed a relatively higher occurrence of six in 2,144,725 (2008-2017). However, given that our patient population consisted of hospitalized patients who were older than the general population on average, implying a sicker cohort, we suspect that this represents an underdiagnosis of cases.

Despite its rarity, AHA is associated with high mortality and resource utilization [1]. As in previous studies, AHA was associated with malignancy and autoimmune disease in our study population. Four patients who received a bypassing agent with IST achieved remission with cessation of bleeding, complete normalization of FVIII levels, and eradication of the factor inhibitor. Despite this high remission rate with the standard of care, four of the five deceased patients had bleeding-associated mortality, pointing to the high relapse rate of this disease. We suspect that this high mortality rate in our cohort compared to the EACH2 cohort is due to the higher median age and markedly higher Bethesda titer, as well as the small sample size in our study. The time from initial bleeding onset to diagnosis was over one week on average, but this was likely affected by one significantly delayed diagnosis in a patient on concurrent warfarin.

Diagnostic delays

A consistent issue spanning multiple decades has been the delays in the diagnosis of AHA, identified by the EACH2 registry, our own findings, and other studies [1,8]. In a study, the time from initial bleeding presentation to diagnosis was two to seven days in 121 patients (25.5%), and the time to diagnosis was greater than seven days in 161 patients (35.3%). Surprisingly, 11.2% of patients were diagnosed more than one month after the presentation. We observed similar diagnostic delays in our patient cohort, with a median time to diagnosis of 14 days. The resulting frailty due to major bleeding emphasizes the importance of achieving hemostasis and eradicating the factor inhibitor in a timely fashion.

Another study reported a 19-day median time to definitive diagnosis in their cohort. The reasons for delays were broad, including laboratory testing delays, lack of recognition of abnormal test results, and minimal hematology consultation. Two confounders, pseudo-thrombosis (i.e., distal extremity hematoma mistaken for thrombosis) and pre-existing anticoagulant therapy, were also noted to contribute to the reported diagnostic delays [12]. There are many roadblocks in the pathway to diagnosis: challenges in differentiating between typical and atypical bleeding, unfamiliarity with AHA and other bleeding disorders, and inappropriate testing hierarchy [12]. The lack of clinical suspicion is a major hurdle that is currently understudied, and an assessment of it may present an opportunity to explore new and innovative methods when evaluating patients presenting with bleeding and bruising.

AHA algorithm

**Figure 1:**
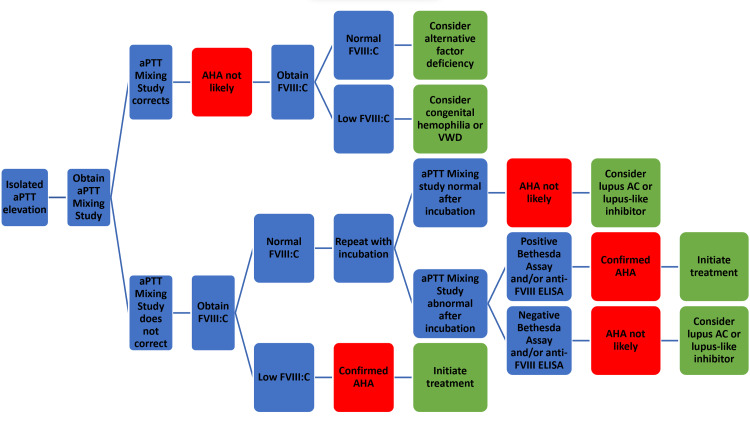
Diagnostic algorithm for unexplained bleeding with isolated prolonged aPTT aPTT: activated partial thromboplastin time; FVIII:C: factor VIII activity; AHA: acquired hemophilia A; VWD: von Willebrand disease; ELISA: enzyme-linked immunosorbent assay; AC: anticoagulant

In light of our findings, we propose an algorithm for the evaluation of unexplained bleeding that could be helpful for frontline practitioners in the diagnosis of AHA and subsequent therapeutic interventions (Figure 1). The initial step is the identification of a patient with new, unexplained bleeding. In the setting of unexplained bleeding, a detailed clinical history, including medication use, along with a thorough physical examination is critical. Prompt primary laboratory testing should include a complete blood count, a metabolic panel including creatinine and bilirubin, and coagulation testing including aPTT and prothrombin time with international normalized ratio (PT/INR). A resulting isolated aPTT elevation will initiate subsequent steps. Early inpatient hematology consultation is recommended. An important point to highlight is that we recommend concurrently ordering an aPTT mixing study and a factor VIII activity (FVIII:C) once a prolonged aPTT is confirmed. This may decrease the time to initiate treatment and improve patient outcomes. If the mixing study result is abnormal with low FVIII:C, hemostatic treatment could be initiated with concurrent confirmatory Bethesda assay or anti-FVIII ELISA, preventing further delay in patient recovery and hopefully reducing potential complications. If there is limited availability of specialty testing or prolonged delays in getting test results, such as for FVIII:C, or an inability to confirm a diagnosis at any stage of the algorithm, transferring the patient to a higher level of care with these laboratory and hematology services should be strongly considered.

The importance of prompt treatment initiation cannot be understated. When the diagnostic delay is greater than one month, there will be a significant increase in the days that the patient is required to be on hemostatic therapy compared to diagnosis before one month (23.8 ± 13 vs. 7.6 ± 5.7 days, respectively; p=0.003) [12]. The interval between initial and final bleeding episodes, including relapses, is also extended when there is a delay in diagnosis of more than a month (49 ± 52 versus 20 ± 20 days, respectively; p=0.05) [12]. There is a promising benefit to beginning therapy in general. In the EACH2 study, of the 474 patients that presented initially with a bleeding episode, 315 patients (66.5%) had only one bleeding episode with no signs of relapse after undergoing successful primary therapy [1].

Bypassing agents and immunosuppressive therapy

Recombinant activated factor VIIa (NovoSeven) is used for primary hemostatic treatment in hemophilia A or B with inhibitors to factor VIII or factor IX, via activation of the extrinsic pathway. In a post-approval study based on the Hemostasis and Thrombosis Research Society Registry (HTRSR), hemostasis in AHA with recombinant activated factor VIIa was achieved at a higher rate as first-line therapy than as second-line therapy (86.6% vs. 66.7%), which was similar to results from the EACH2 study (91.2% vs. 79.4%) [5,13]. For initial treatment, a bolus injection of 90 ug/kg every two to three hours is recommended until hemostasis is achieved [8].

Activated prothrombin complex concentrate (FEIBA) was originally approved for congenital hemophilia with inhibitors. This preparation contains clotting factors (non-activated II, IX, X, activated VII) that generate thrombin bypassing the deficient or neutralized factor VIII levels. Its use has been expanded for the prevention and treatment of bleeding episodes in AHA [8,9]. In a small pivotal study, all seven patients with AHA, initially treated with high-dose activated prothrombin complex concentrate followed by post-hemostasis reduced-dose activated prothrombin complex concentrate as maintenance therapy until FVIII inhibitor titer was less than 50%, experienced no recurrence of bleeding. This was compared to a historical control group of 11 subjects in which six patients experienced bleeding recurrence when treated only during acute bleeding. There are no systematic reviews on the medication, but the EACH2 registry has reported greater than 90% bleeding control when it is used as first-line. Its current dosing recommendation is a bolus injection between 50-100 U/kg every 8-12 hours, not exceeding 200 U/kg/day [8].

Recombinant porcine sequence FVIII (Obizur) was approved for bleeding episodes in patients with AHA. In a prospective single-arm clinical trial, 24 of 29 patients achieved greater than 80% FVIII:C with initial dosing of 200 U/kg [6]. Due to the remarkably high success levels of FVIII, subsequent observations have demonstrated similar efficacy at a lower dosage of 100 U/kg [6,11]. One aspect that distinguishes Obizur from other bypassing agents is the ability to monitor FVIII activity, thereby allowing regimens to be tailored to individual patients [6]. The manufacturer’s recommended dosing for initial treatment remains 200 U/kg followed by subsequent dosing with a goal of maintaining FVIII:C activity >50%. Continuous FVIII:C monitoring is needed to prevent markedly elevated FVIII:C, which may increase the risk of thrombosis [11]. 

There is a concurrent approach to bleeding cessation and eradication of the FVIII inhibitors. The intention is to avoid causing a delay in inhibitor removal, and hence initiating immunosuppressants while attempting to stop the bleeding is critical. Some studies have demonstrated efficacy via plasma exchange [14]. Otherwise, the focus has been on IST, starting with corticosteroids such as prednisone and prednisolone, with the subsequent addition of cyclophosphamide, azathioprine, vincristine, and mycophenolate mofetil. Other IST agents used have included intravenous immunoglobulin (IVIG), cyclosporine, and rituximab [8].

First-line IST for AHA inhibitor eradication currently entails a combination of prednisone (1 mg/kg/day) with or without oral cyclophosphamide (50-100 mg/day). The EACH2 study showed a 70% vs. 48% complete remission (CR) frequency when treated with combination therapy vs. steroid monotherapy, respectively. Rituximab-based regiments in the EACH2 study obtained 59% CR, and 50% achieved CR after failure with other first-line regimens or relapse. However, there was no difference between therapies in terms of sustained remission and survival. Non-response to first-line therapy was defined as the failure of FVIII:C to increase, and the inhibitor titer not declining after three to five weeks of treatment. Second-line alternatives to rituximab include cyclosporine, tacrolimus, and mycophenolate mofetil. IVIG has not been shown to effectively alter inhibitor levels [15].

So far, neither the initial studies nor propensity score-matched analyses of registry data on bypassing agents have demonstrated superior safety or efficacy of any specific bypassing agent over the others [8]. All the larger prospective cohort studies examined patients between 2001 and 2013, and, while some included patients on modern bypassing agents including recombinant activated FVIIa, recombinant porcine sequence FVIII, and activated prothrombin complex concentrate, they may not have been available to all patients [1-6]. Furthermore, dosing standards and IST practices have changed since those studies were performed, yet a multi-arm randomized controlled trial comparing bypassing agents and IST would come up with an optimal sequence of therapies for better patient outcomes.

Therapies currently under study

Since the EACH2 database study, only one new bypassing agent has been approved, another recombinant FVIIa (Sevenfact®, HEMA Biologics, Louisville, KY), and there are ongoing studies involving other agents. Off-label uses for other therapies are also being evaluated [13,16,17].

One such drug is emicizumab (Hemlibra®, Genentech, South San Francisco, CA), a bispecific, humanized, recombinant monoclonal antibody that mimics FVIII by concurrently binding factor IXa and factor X. It is approved for congenital HA with particular benefit in patients with FVIII inhibitors [18]. A recent study evaluated emicizumab in 12 patients with AHA and there were no deaths from bleeding or thrombosis in the cohort [19]. However, other studies have reported thrombosis-related deaths in those using emicizumab for AHA with inhibitors [20]. Because of the mixed results on its efficacy and a history of severe adverse effects, emicizumab has not yet entered mainstream practice or guidelines for AHA and it remains unclear if it can be used in lieu of bypassing agents as first-line therapy.

Another drug that has been used off-label is bortezomib (Velcade®, Takeda Pharmaceutical), a proteasome inhibitor primarily used for multiple myeloma and mantle cell lymphoma. Its intended use in AHA is for inhibitor eradication. Although no clinical trials in patients with AHA have been completed to date, case reports have demonstrated a positive response to this drug [21].

In cases of severe bleeding, markedly elevated inhibitor titers, or disease refractory to routine IST, immunoadsorption (IA) has been considered as an adjunctive therapy. A systematic review and meta-analysis of 106 patients with AHA receiving IA concluded that patients receiving IA had better overall survival and remission rate outcomes compared to the historical EACH2 cohort [1,22]. Risks related to IA include infection risk due to the use of a central venous catheter, hypocalcemia, bleeding due to clotting factor depletion from apheresis, and limited availability. However, due to the high cost of bypassing agents, IST, and associated care, IA may still be a cost-effective option, though there have not been any recent studies on the therapy [23].

Study limitations

This study has some limitations, primarily its single-center, retrospective design. All the data were collected retrospectively. While outside records were reviewed, not all outside records may have been shared, and hence not all bleeding events may have been recorded. Given the small sample size, formal statistical analysis could not be performed. Additionally, data on the time to recurrence of low FVIII was not collected.

## Conclusions

Patient characteristics, management, and outcomes in our cohort were similar to the findings described in prior studies, including EACH2. Considerable delays were observed in the time from the initial presentation of atypical, prolonged, or profuse bleeding symptoms to the diagnosis and treatment of AHA. These delays have been reported in some other studies as well, and they present a significant dilemma. Given the high mortality rate associated with AHA, delays in diagnosis and treatment may lead to more protracted bleeding. It is important to note that no correlation was found between diagnostic delays and responses to any particular treatment regimens in the EACH2 database. However, a more prompt and accurate diagnosis of AHA will likely lead to less morbidity and mortality among patients with AHA. We also suspect that Neyman Bias may contribute to underdiagnoses of AHA due to the rapid decompensation with major bleeding, meaning that some patients developing factor inhibitors may have died before a full hematologic evaluation could be performed.

Our algorithm for the evaluation of unexplained bleeding is intended to reduce diagnostic delays and circumvent a heuristic approach to the diagnosis of AHA. It is based on current hemostatic treatment modalities, as well as therapies currently in development. We hope this will guide community practitioners in the management of patients with unexplained bleeding through prompt diagnosis and treat AHA effectively.
